# Animated stories of medical error as a means of teaching undergraduates patient safety: an evaluation study

**DOI:** 10.1007/s40037-019-0498-1

**Published:** 2019-02-14

**Authors:** Kerri Cooper, Emma Hatfield, James Yeomans

**Affiliations:** 1 Department of Surgery and Cancer, Imperial College London, London, UK; 2 Imperial College Healthcare NHS Trust, London, UK

**Keywords:** Patient Safety Education, Medical Error, Medical Animation, Storytelling

## Abstract

**Background:**

Storytelling is a powerful form of communication which can improve attention and lead to lasting behavioural changes. Addressing the need to incorporate patient safety teaching into undergraduate medical curricula, it was hypothesized that medical students could benefit from hearing clinician stories of medical error. The medium of animation was considered to be a potentially engaging means of presenting stories of error to a large audience.

**Methods:**

Three animated videos were developed to accompany audio recordings of junior doctors describing their experiences of a serious incident or near-miss event. The videos were delivered to 200 final-year medical students with a subsequent large-group discussion directed at understanding contributory factors. An evaluative questionnaire exploring learners’ reactions and modification of beliefs and perception was distributed. The questionnaire included questions rated on a modified Likert scale and a free-text box. A mixed-methods analysis was conducted with descriptive statistics and qualitative content analysis of the free-text responses.

**Results:**

Of the 200 students who attended, 104 completed the questionnaire and 83 completed free-text feedback. Most students responded positively to hearing stories of medical error and felt that the animated videos improved their engagement while the voice recordings helped bring the cases to life. The majority of students agreed the session would impact on their future practice.

**Conclusion:**

This pilot study confirmed that undergraduate students consider animated, personal stories of medical error an effective, engaging means of learning about patient safety. Longitudinal studies are required to confirm if measurable behaviour change is achieved.

## Background

The need to emphasize patient safety within undergraduate medical curricula has been advocated by regulatory bodies across the world, including the World Health Organization [1]. Yet, a recent systematic review of undergraduate curricula found a lack of research addressing teaching interventions in this area [2].

To date, efforts to improve patient safety education have tended to adopt a systems-level approach [3]. In a professional context, some redress to this ‘top-down’ approach has materialized with the dissemination of Schwartz Centre Rounds, whereby healthcare providers share experiences from difficult cases to enhance the personal connections they make with colleagues and patients [4]. However, the use of storytelling for the specific purpose of disseminating learning from medical error remains underexplored, representing a significant missed learning opportunity as evidence from the field of applied psychology suggests learning from stories of error is more effective than learning from perfect examples [5].

A purely systems-based approach to medical error also discounts the potential for narrative to effect desirable behaviour change. Evidence from social sciences supports the power of stories in challenging preconceived beliefs versus the use of statistics alone [6] and mounting evidence has demonstrated the successful application of ‘narrative interventions’ to effect health behaviour changes [7]. One article describes how in a sample of 404 ‘college-age’ women, those exposed to expert-peer narrative videos about human papilloma virus vaccination were twice as likely to have sought out vaccination at 2 months compared with controls [8]. Its application in healthcare education has also been documented; a systematic review of digital storytelling for healthcare professions finds an ‘eclectic’ range of purposes for this approach, with the majority of studies identified applied in a nursing context [9]. This review found evidence of self-reported learning and behaviour change but highlighted the need for further research in the use and impact of digital storytelling in healthcare education and noted a minority of studies explored this approach in medical cohorts [9].

This study sought to explore innovative means of disseminating experiential insights from cases of medical error to undergraduates in a ‘Storytelling’ format. Owing to shame often associated with medical error, [10] it was recognized it would be challenging to recruit speakers. As such, the medium of animation was considered; educational research has highlighted its capacity to ‘allow visualization of subjects and scenes that could never be captured on camera’ [11].

It was hypothesized stories of medical error could facilitate learning about patient safety and that animated videos could allow a large audience to be reached whilst promoting engagement.

The primary objective was to evaluate to what extent hearing personal stories of medical error impacts on medical students’ reactions, perceptions and beliefs about patient safety, with a secondary objective to evaluate the impact of animation on communicating stories of medical error to medical students.

## Method

### Teaching session design and delivery

A storytelling teaching session using animation was designed by an undergraduate teaching fellow with training in medical education and mentorship, overseen by a medical consultant acting as the Director of Clinical Studies for undergraduate medical students at Charing Cross Hospital, London.

The session was included in a transition course that was delivered to final year medical students at Imperial College London, who had recently completed their final year exams.

**Fig. 1:**
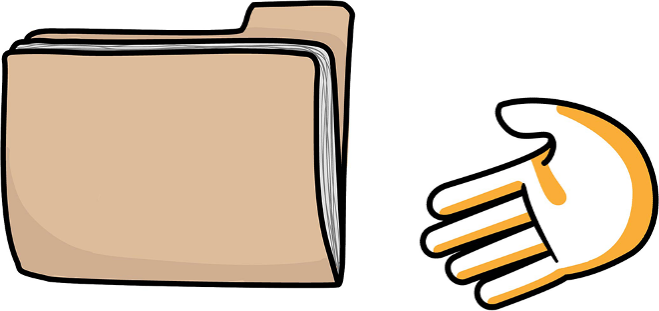
Example animation: Depicts incorrect handover of the documented plan [12]

Prior to the session, three junior doctors were audio recorded describing incidents they had been involved in where a patient was harmed or experienced a near-miss event. They described the patient journey, their own involvement with the case and a short reflection. Animated videos were then developed to accompany the voice recordings, depicting salient points from each narrative in coloured, moving two-dimensional images (Fig. 1) using *Videoscribe* software [12]. No patient identifiable data were included, and videos were stored within a secure password-protected folder.

**Fig. 2:**
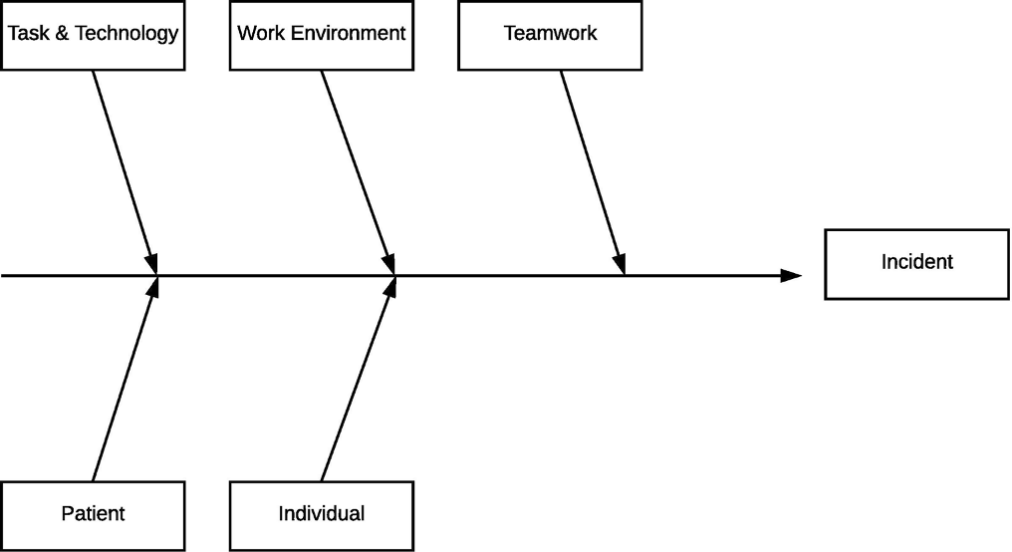
Fishbone analysis for healthcare [13]

A total of 200 final year medical students attended two separate, identical sessions, with 100 students per session; 111 (56%) students were female, 89 (44%) were male. Subsequent to each animated story being played, the two faculty members who had designed the course facilitated a large-group discussion. A fishbone analysis tool (Fig. 2; [13]) was used to guide identification of factors contributing to each case.

### Evaluation

An evaluation questionnaire was developed to accompany the session. It was decided that evaluation should be directed at ‘learner’s reactions’ and ‘modification of perceptions and beliefs’ as described in modified Kirkpatrick’s four levels of evaluation [14]. The questionnaire consisted of questions where respondents were asked to indicate on a modified Likert scale (1: strongly disagree, 2: disagree, 3: neither agree nor disagree, 4: agree, 5: strongly agree) the degree to which they agreed with statements pertaining to the session. In addition, a free-text box was included for further comments.

Prior to delivering this teaching session, a version was trialled on the committee who organized the transition course. The committee consisted of nine full-time medical educators—all of whom were undertaking formal education qualifications. Informal feedback was sought from the committee about the session and, upon review, alterations were made to wording and inclusion/exclusion of questions within the questionnaire.

Participants were advised data collected may be used for publication. The institution’s Medical Education Ethics Committee confirmed that ethical approval was not required for an evaluation of teaching.

### Data analysis

A mixed-methods analysis was performed. Results from the questionnaire were subject to descriptive statistics. Thematic analysis was applied to the qualitative dataset. Two investigators conducted descriptive coding of the dataset to derive core domains. Within these core domains, in vivo coding was conducted. At each stage of coding, investigators maintained notes and met to resolve any conflicts in interpretation of the data.

## Results

A total of 104 students out of the 200 who attended the sessions completed the evaluative questionnaire and 83 provided free-textbox feedback.

### Quantitative analysis

Of the respondents, 97 (94%) agreed/strongly agreed that completing the storytelling session would impact on their practice as doctors and 91 (88%) agreed/strongly agreed they would benefit from similar sessions in future, the remainder of respondents disagreed/strongly disagreed with both these statements. Out of the 104 participants, 99 (96%) agreed/strongly agreed that hearing doctors describe incidents they had been involved in was a compelling way to learn about patient safety, 5 (4%) respondents were neutral on this point. In response to the statement, ‘the animations positively impacted on my understanding of the case’, 97 (94%) agreed/strongly agreed and 7 (6%) disagreed/strongly disagreed, while 104 (100%) of students agreed/strongly agreed that the animated videos were more engaging than a PowerPoint presentation format. Regarding the fishbone analysis tool, 91 (88%) participants agreed/strongly agreed it was helpful in understanding the cases while 11 (11%) were neutral and 2 (1%) strongly disagreed.

### Qualitative analysis

Of 83 participants who completed free-text feedback, 78 provided positive feedback on the session. Feedback was directed at three core areas which included content, impact of the animated videos and overall delivery of the session.

### Content

Key themes that emerged were *open discussion, reflection, case number* and the *proximity of experience*. Learners appreciated open discussion of mistakes in medicine, finding this reassuring: ‘*It was helpful and reassuring to hear doctors discuss mistakes honestly and openly’ *(Participant # 40). Although one participant described the cases discussed as ‘scary’.

Learners appreciated that the cases provoked reflection and valued hearing the doctors involved in the cases discuss their own reflections: *‘I think it’s such a good thing to encourage sharing and reflection on when things go wrong, as there will always be something to learn from it which will help improve patient safety’ *(Participant # 12).

There was some discrepancy in attitudes to the ideal number of cases to include with some learners commenting positively, whilst others would have preferred a greater number. Finally, while some learners appreciated hearing about cases from relatively near-peers, others would have preferred a session focused on doctors in their first year of training.

### Impact of animated videos

The key themes included *engagement* and *depiction of ‘real life*’. The majority of participants found the animated videos improved their engagement with the session: *‘Really enjoyed the use of animation’ *(Participant # 57), with just one participant describing the videos as ‘distracting’. Participants appreciated hearing the voices of the doctors describing the cases as it helped bring the cases to life: *‘Liked the voiceover to make real’ *(Participant # 4).

### Delivery

Key themes to emerge included *group size* and *facilitated discussion*. Participants largely would have preferred these sessions to be conducted in small groups and expressed a preference for guided group discussion over unstructured group discussion: *‘I think this session was very informative and helpful but maybe would have been better suited to a small-group format to facilitate more in-depth discussion.’ *(Participant #70).

## Discussion

Mirroring disparate applications of allegorical approaches in healthcare, [7, 8] this study confirmed that undergraduate learners respond positively to personal stories of medical error, as a means of learning about patient safety. The use of animation served to enhance engagement and comprehension.

Key benefits demonstrated of the storytelling approach included its ability to engage learners and its potential to impact behaviours. These findings may be explained by Rose et al.’s [15] conclusions, in their meta-narrative review of storytelling in healthcare, that stories aid sense-making and ‘are uniquely primed to elicit empathy and compassion.’ The emotional impact of the stories may be key to their influence. A literature review examining the role of emotion in learning and transfer of clinical skills found ‘emotion influences how individuals identify and perceive information, how they interpret it, and how they act on the information’ [16]. Similarly, a review of digital story-telling in healthcare details examples where digital stories engender empathy to a particular subgroup and alter attitudes and behaviours [9]. The authors of this review describe examples of co-creation of stories between healthcare professionals and patients, [9] and particularly in the setting of patient safety, the inclusion of patient voices may enrich narrative-based learning.

As with practising clinicians, [10] an important finding from the study was the positive response of medical students to open, constructive discussion of error. Exposing students to clinicians speaking about mistakes may stand to enculture an attitude of openness before entering clinical practice where students risk acquiring negative behaviours for dealing with error [17]. Again, the narrative approach, using near-peers, may have heightened the impact of discussing and reflecting on error by facilitating a form of role-modelling [18]. Small group sizes, skilled facilitated discussion and use of near-peer experiences may help ensure such sessions achieve reflection and learning.

As demonstrated in this study, animation videos represent a powerful medium for communicating stories of medical error to wider audiences. Removing the need for the presence of a clinician circumvents the challenge of recruiting speakers from within a culture that is struggling to enact principles of candour [10] and may allow greater openness and more objective deconstruction of cases of medical error. One key advantage of using animation was its ability to engage learners and aid comprehension. Within the literature, studies have mainly focused on animation as a tool for patient education and have been shown to be an effective means of distilling complex information [19, 20]. For example, Hermann [20] randomized patients undergoing thyroidectomy to either receive written information or a 3D computer animation, prior to the surgery, explaining the procedure and possible complications. Understanding of the procedure and complications as well as trust in the treatment was significantly better in the group who watched the animation [20]. Analogously, the positive response to the animated videos in this study may reflect their ability to encourage learners to visualize the complexities of the clinical environment.

### Limitations

This study focused on self-reported attitudes; further longitudinal and comparative studies would be required to establish whether similar sessions could result in measurable behaviour change.

## Conclusion

In contrast to a dominant focus on systems-level teaching in patient safety, this evaluative study confirmed that undergraduate students consider personal stories of medical error from near-peers an engaging and effective means of learning about patient safety. The use of animation served to enhance the storytelling approach. Further exploration would need to be undertaken to examine if similar sessions could improve safety behaviours in practice.
